# Evaluation of a school-based participatory intervention to improve school environments using the Consolidated Framework for Implementation Research

**DOI:** 10.1186/s12889-021-11644-5

**Published:** 2021-09-03

**Authors:** April K. Wilhelm, Maria Schwedhelm, Martha Bigelow, Nicole Bates, Mikow Hang, Luis Ortega, Shannon Pergament, Michele L. Allen

**Affiliations:** 1 Program in Health Disparities Research, Department of Family Medicine and Community Health, University of Minnesota, 717 Delaware St. SE, Suite 166, Minneapolis, MN 55414 USA; 2 Department of Curriculum and Instruction, College of Education and Human Development, University of Minnesota, Peik Hall, 159 Pillsbury Drive SE, Minneapolis, MN 55455 USA; 3 ESTEM, Office of Equity, Saint Paul Public Schools, 600 Weir Drive, Woodbury, MN 55125 USA; 4 SoLaHmo Partnership for Health and Wellness, Inc, Community University Health Care Center, 2001 Bloomington Avenue, Minneapolis, MN 55404 USA

**Keywords:** CFIR, Participatory research, Implementation, Qualitative methods

## Abstract

**Background:**

Participatory research offers a promising approach to addressing health inequities and improving the social determinants of health for diverse populations of adolescents. However, little research has systematically explored factors influencing the implementation of participatory health interventions targeting health disparities.

**Objective:**

This study examined the utility of the Consolidated Framework for Implementation Research (CFIR) in identifying and comparing barriers and facilitators influencing implementation of participatory research trials by employing an adaptation of the CFIR to assess the implementation of a multi-component, urban public school-based participatory health intervention.

**Methods:**

We collected qualitative data over a one-year period through weekly team meeting observational field notes and regular semi-structured interviews with five community-based participatory researchers, one school-based partner, and four school principals involved in implementing a participatory intervention in five schools. Adapted CFIR constructs guided our largely deductive approach to thematic data analysis. We ranked each of the three intervention components as high or low implementation to create an overall implementation effectiveness score for all five schools. Cross-case comparison of constructs across high and low implementation schools identified constructs that most strongly influenced implementation.

**Results:**

Ten of 30 assessed constructs consistently distinguished between high and low implementation schools in this participatory intervention, with five strongly distinguishing. Three additional constructs played influential, though non-distinguishing, roles within this participatory intervention implementation. Influential constructs spanned all five domains and fit within three broad themes: 1) leadership engagement, 2) alignment between the intervention and institutional goals, priorities, demographics, and existing systems, and 3) tensions between adaptability and complexity within participatory interventions. However, the dynamic and collaborative nature of participatory intervention implementation underscores the artificial distinction between inner and outer settings in participatory research and the individual behavior change focus does not consider how relationships between stakeholders at multiple levels of participatory interventions shape the implementation process.

**Conclusions:**

The CFIR is a useful framework for the assessment of participatory research trial implementation. Our findings underscore how the framework can be readily adapted to further strengthen its fit as a tool to examine project implementation in this context.

**Supplementary Information:**

The online version contains supplementary material available at 10.1186/s12889-021-11644-5.

## Background

Participatory approaches to research such as community-based participatory research (CBPR) and participatory action research (PAR) have proven effective in guiding public health intervention design in various contexts, particularly for interventions addressing health inequities [1, 2]. However, the utilization of theoretical frameworks to guide the development [3] or the systematic project implementation assessments of participatory interventions [4–7] is in its infancy. Participatory interventions are characterized by distinct practices that may influence implementation including shared decision-making processes, the iterative nature of intervention development and implementation, and an explicit commitment to accommodating local stakeholder perspectives and priorities [8]. Understanding specific implementation facilitators and barriers within the participatory intervention context is essential for improving scientific and community impacts [9].

The Consolidated Framework for Implementation Research (CFIR) is an interdisciplinary theoretical framework composed of 39 constructs organized within five overarching domains (i.e., intervention characteristics, outer setting, inner setting, characteristics of individuals, and the process of implementation) [10]. In the CFIR, Damschroder et al. [11] melded theories from a range of disciplines to enable the systematic assessment of potential facilitators and barriers to intervention implementation effectiveness. While initially applied within behavior change interventions in healthcare settings [12–14], the CFIR has been adapted for use in public health [7, 15] and school settings [16, 17] and has undergone multiple methodologic adaptations [14, 18].

Several studies have begun to employ the CFIR to assess participatory intervention implementation in a variety of clinical [5, 6] and public health settings [7]. However, these initial studies were small and did not explicitly examine the CFIR’s fit for assessing the implementation of participatory research trials, defined as trials that combine “the grounding of community priorities and insights offered by participatory research with the evidence-generating capabilities of formal trials” [8]. Previous participatory intervention implementation assessments indicate that several CFIR constructs may be particularly important in participatory trial implementation. While the CFIR doesn’t explicitly account for how to incorporate stakeholder perspectives and goals in a participatory implementation process [5], collaboration with stakeholders, defined as individuals within and/or external to the organization who are involved with implementation, can inform implementation plans, broadly increasing engagement and strengthening the intervention’s execution along with its relevance and acceptability for participants [19]. Additionally, participatory interventions’ inherent adaptability may augment their compatibility and implementation across a range of institutions [3, 20]. Given the need for ongoing partnerships between institutional leaders and other stakeholders within participatory interventions and the potential for challenges in maintaining these partnerships [21], leadership engagement is also likely to be influential.

This study aimed to examine the utility of the CFIR for identifying implementation facilitators and barriers within participatory research trials. To do this, we apply an adaptation of the CFIR to one participatory research trial to identify and compare barriers and facilitators influencing the variation of the multi-component public health participatory intervention’s implementation in school settings.

### Intervention

Project TRUST (Training for Resiliency in Urban Students and Teachers), hereafter referred to as TRUST, is a three-component, five-year pragmatic participatory intervention in ten middle and high schools in an urban school district. TRUST aims to address school connectedness as a means to improve behavioral health [22, 23] and academic outcomes [22, 24] for Black, Indigenous, People of Color (BIPOC) students in particular. BIPOC students experience barriers to building connections to their schools and teachers that can disrupt the protective benefits of these social determinants [25–27].

TRUST was implemented in two waves to create a delayed control group for assessing the pragmatic trial. The first five schools (four middle and one high school) implemented TRUST between July 2017 and June 2018 and are this paper’s focus. All enrolled schools were diverse in terms of race/ethnicity, with approximately 80% students of color, including 29% Asian, 18% Latino, 26% African or African American, and 2% Indigenous.

TRUST intervention components are 1) youth participatory action research (YPAR) generated recommendations to address connectedness, 2) parent participatory action research (PPAR) generated recommendations, and 3) a teacher professional development (PD) curriculum. Ten YPAR researchers, (two from each school), and seven PPAR researchers (one to two from each school) participated in Wave 1. PAR researchers represented the range of diverse identities (e.g., age, gender, race/ethnic, faith, and immigrant) within their schools, which varied by site. The majority were new to action research and did not have prior relationships with school administrators. Teams spent the year prior to implementation conducting PAR; they framed the research questions and methodologic approaches, collected and analyzed data, and formulated policy, practice, and procedural recommendations in their schools to enhance student connectedness. PAR teams were tasked with identifying three to five action recommendations to share with their respective school leadership teams for implementation (Table 1); all principals committed to implementing three YPAR and three PPAR recommendations when they agreed to participate in this study. The third component, a nine-session PD curriculum, focused on enhancing teacher knowledge and skills in building trusted relationships with BIPOC students using a positive youth development framework.

**Table 1:** Description of Project TRUST intervention components by school

School number	YPAR action recommendation examples	PPAR action recommendation examples
1	•More opportunities to learn and share across cultures •Additional class options including art	•Host formal parent gatherings in school three times per year •Increase parent-school communication on bullying
2	•Develop counseling opportunities for students who are bullying or being bullied •Increase hallway regulation to prevent physical fights	•Examine use of tablets, especially parental awareness and ability to regulate tablets at home •Provide opportunities for families to help work through student-staff conflicts
3	•Increase mainstream teacher training for work with English language learner (ELL) students •Hire more ELL teacher staff	•Host a series of stakeholder dialogues to discuss safety related to bullying •Increase and improve the quality of communication between school and non-English-speaking parents
4	•Increase teacher training on how to talk about race with their students •Create an in-school suspension space to help students process their frustrations	•Improve school-parent communications about expectations and use of school technology platforms •Enhance sixth-grader transition using an existing leadership program
5	•Transform the student behavioral referral process •Hire more staff of color	•Develop a parent buddy system and open house to welcome new families each fall •Recruit an outreach coordinator who is also a parent of color

### Implementation team

The TRUST core team is an academic-community partnership that includes members from a United States Midwestern university, a community organization aimed at using CBPR to improve the health of marginalized communities, and an urban school district. TRUST’s core team members facilitated the PAR process in the year prior to implementation. During the implementation year, TRUST core team members coordinated meetings between school administrators and PAR researchers in each school to develop an implementation plan. A subset of PAR researchers also worked with TRUST core team members to facilitate the implementation process. For example, PPAR researchers from several schools collaborated with the TRUST team to address PPAR action recommendations (Table 1) by engaging parents and fostering community through an Intentional Social Interaction implementation modality, a practice-based CBPR engagement model developed by Marnita’s Table where participants meet over a meal to catalyze connection and collaboration on important public policy issues [28]. Finally, TRUST core team members prepared and delivered the teacher PD sessions in schools.

## Methods

### Study design

We conducted a longitudinal qualitative study to assess TRUST implementation in Wave 1 schools. Using a largely deductive analytical approach driven by the CFIR following previously described methodologies [12], we assessed how particular constructs manifested in each school’s implementation experience and employed a deliberative consensus process to rate each construct by facility. We then looked at construct ratings across facilities to identify which constructs distinguished between low and high implementation schools. Given that all study data was collected from research team members and collaborators as part of a program evaluation, this study protocol was not considered to be human subjects research and was exempted by the authors’ Institutional Review Board. This therefore omitted the need for obtaining consent when collecting data. All components of the Standards for Reporting Qualitative Research [29] are addressed in this manuscript.

### Study participants

Study participants included six TRUST core team members involved in implementation, five administrators from four of the five Wave 1 schools who served as the primary points of contact for implementation, and ten youth and seven parent PAR researchers. The need for obtaining consent was waived by the study’s Institutional Review Board as above. However, all participants verbally consented to participate in this study and did not receive participation incentives.

### Data collection

Two TRUST team members not involved in implementation collected longitudinal qualitative data from July 2017 to June 2018 (the Wave 1 implementation year). Triangulated data sources and methods included weekly observational field notes of research team meetings, semi-structured interviews with TRUST core team members and school administrators, and PAR researchers’ written perspectives and assessments on implementation processes. Regular semi-structured interviews with core team members probed for specific barriers and facilitators to implementation activities (Supplemental File 1). Interviews ranged in frequency from weekly to monthly based on the team members’ level of involvement with daily research activities. A.W. conducted all face-to-face and phone TRUST core team interviews and took non-verbatim notes to create written transcripts. School administrators from three schools and one district-level school administrative partner on the TRUST team participated in one 60-min in-person, audio-recorded interview at the end of the implementation year that were transcribed verbatim. Administrator interview questions developed for this study were guided by CFIR constructs with a focus on intervention characteristics and inner and outer settings (Supplemental File 2). One administrator elected to respond to interview questions through email while two administrators at another school participated in their interview simultaneously. One administrator left their school prior to the exit interviews and did not respond to outreach efforts.

### Data analysis

#### Qualitative coding

We used directed content analysis [30] to deductively code our longitudinal data sources using the CFIR as a coding framework. Prior to coding, we adapted the CFIR to more closely align with our PAR approach by selecting 31 relevant constructs and revising their names and definitions through a consensus-driven discussion (Table 2). For example, we subdivided networks and communications into quality of formal communications and social capital sub-constructs to disentangle the influences of these related but distinct concepts. We remained open to new concepts within the data that did not fit within the CFIR, inductively coding these concepts and ultimately incorporating three new sub-constructs into our adapted framework. A.W. and M.S. independently coded a subset of the transcripts using a line-by-line coding approach and then compared coding and discussed discrepancies prior to assigning final codes and completing the coding process with the remaining data sources. Next, these two authors developed a summary memo for each school, selecting representative excerpts for each construct following the approach described by Damschroder and Lowery [12]. The team used the software program Dedoose [32] to organize and manage data sources.

**Table 2:** CFIR construct definition adaptations for Project TRUST organized by domain^a^

CFIR Construct	TRUST operationalization of construct definition
I. Intervention Characteristics	
**Adaptability**	Degree that participatory processes allowed for intervention adaptations to meet stakeholder needs
**Complexity**	Perceived complexity and flexibility of the TRUST intervention components (i.e., professional development, YPAR, and PPAR)
General Intervention Logistical Complexity^b^	Perceived complexity and flexibility of the TRUST research and evaluation components
**Design Quality & Packaging**	Perceived quality in how TRUST was presented in terms of materials, process, and potential impact/return on investment
II. Outer Setting	
**Participant Needs & Resources**	Extent to which school/district understands and is oriented to the needs and preferences of students
**Cosmopolitanism**	Degree to which school leadership is networked with other schools and/or community organizations
**External Policy & Incentives**	External mandates that exerted pressure on schools to participate in TRUST (e.g., school improvement status and other federal, state or district policies)
III. Inner Setting	
**Social Structural Characteristics**	School contextual and social organizational components, such as demographics, school structure, degree of staff turnover, and concentration of decision-making autonomy
**Networks & Communications**	Degree to which people involved in TRUST had strong working relationships
Quality of Formal Communications^c^	The nature and quality of formal and informal communications within the school and between TRUST and the school
Social Capital^c^	The quality and the extent of relationships within schools and across partnering organizations
**Culture**	School culture regarding student and parent voice
**Implementation Climate**	A school’s capacity to change school practices and/or procedures
Tension for Change	Degree to which leaders see the identified issues as problematic and their openness to address them
Compatibility	Alignment with leadership beliefs about how to address the proposed recommendations, and how TRUST fits with existing school workflows and systems
Relative Priority	Importance of TRUST in comparison to other initiatives
Organizational Incentives & Rewards	Extrinsic incentives that TRUST offered for participants (e.g., awards, salary, performance reviews, stature, respect)
**Readiness for Implementation**	Indicators of school’s commitment to implement TRUST
Leadership Engagement	Commitment, involvement, and accountability of those in school leadership roles with TRUST components
Available Resources	Level of resources within the schools themselves that can be dedicated for TRUST implementation and ongoing operations
Access to Information & Knowledge	Ease of access of school members without direct TRUST affiliation to digestible information about TRUST and how to incorporate it into work tasks
IV. Characteristics of Individuals	
**Knowledge & Beliefs About the Intervention**	School leadership and staff familiarity with, attitudes toward, and value placed on TRUST
**Agency**^d^	School leadership and staff socioculturally-mediated capacity to implement TRUST components
**Individual Stage of Change**	Stage of change of school leadership and staff as they progress toward skilled, enthusiastic, & sustained use of the intervention
**Individual Identification with the Organization**	How school leadership and staff perceive their relationship and degree of commitment to their school
V. Process	
**Planning**	Degree to which implementation methods were developed in advance and the quality of these methods
**Engaging**	Degree to which appropriate individuals inside and external to the school were attracted to and involved with TRUST implementation
Opinion leaders	Individuals in the school who had formal or informal influence on the attitudes and beliefs of their colleagues relative to TRUST implementation
Formally Appointed Internal Implementation Leaders	Individuals within the school who were formally appointed for implementing TRUST
Champions	Individuals who dedicated themselves to supporting, marketing, and driving through TRUST implementation
External Change Agents	Individuals affiliated with an outside entity that formally (& positively) influenced or facilitated TRUST implementation decisions
**Executing**	Degree to which TRUST implementation was carried out according to plans
**Reflecting & Evaluating**	Degree to which participants debriefed throughout TRUST implementation as a means of promoting shared learning and improvements

Bolded text indicates CFIR constructs within each domain; plain text denotes sub-constructs

*YPAR* Youth Participatory Action Research

*PPAR* Parent Participatory Action Research

^a^Adapted from original CFIR construct definitions developed by Damschroder et al. 2009

^b^New sub-construct added to the framework

^c^Sub-construct developed from original framework construct definition

^d^Construct redefined as “the socioculturally mediated capacity to act” [31] to draw attention to contextual factors that influence an individual’s belief in their own capabilities to execute action within a participatory intervention

#### Intervention implementation assessment

TRUST core team members collectively ranked the intervention implementation effectiveness within each participating school to reflect the school’s level of implementation of each of the three main intervention components as follows. After reviewing written implementation assessments from PAR researchers, school administrator interviews, and interviews with PAR and PD facilitators, core team members used a consensus-based approach to assign scores for the uptake of each action recommendation and the PD. The two schools with the lowest TRUST uptake were classified as low implementation schools, whereas the two schools with the highest uptake were classified as high implementation schools. We classified the remaining school as an intermediate implementation school because it exhibited examples of both low and high TRUST uptake.

#### CFIR construct rating

We employed a deliberative consensus process incorporating components of consensual qualitative research methods [33] in our multi-stage rating process, including using several judges throughout the analysis to foster multiple perspectives and consensus discussion to assign meaning to the data. Using previously established criteria and methods [12], A.W. and M.S. independently assigned a rating to each construct for each of the five schools that reflected the valence (positive or negative influence) and the magnitude of each construct based on the summary memos (Table 3). We characterized constructs as missing when they lacked adequate data to discern a pattern; constructs that were missing data for all five schools were removed from our analysis, leaving 30 constructs. A.W. and M.S. next met with a subset of the core research team to guide a consensus-driven discussion about each construct to achieve a common understanding of the coding classifications and to agree on a final rating assignment. The small group routinely brought back questions to the entire core research group for clarifying conversations to achieve consensus, contributing to a final three-level consensus process and thus increasing the face validity of the ratings.

**Table 3:** CFIR construct rating assignment criteria^a^

Rating	Criteria
−2	• The construct (or its absence) is a negative influence in the school generally, an impeding influence on work processes, and/or an impeding influence on implementation efforts. • Two or more interviewees described explicit examples of how aspects of the construct manifested negatively.
−1	• The construct (or its absence) is a negative influence in the school generally, an impeding influence on work processes, and/or an impeding influence on implementation efforts. • Interviewees made general statements of how the construct manifested negatively without concrete examples; and/or • There is a mixed effect of different aspects of the construct with an overall negative effect; and/or • Sufficient information exists to indirectly infer a generally negative influence.
0	The construct has a neutral effect in the school, on work processes, and/or on implementation efforts if: • Interviewees provided purely descriptive or generic data without evidence of positive or negative influence; and/or • Interviewees contradicted one another; and/or • Positive and negative influences at different levels in the school balance each other out. We defined this last category as 0 (mixed).
+ 1	• The construct is a positive influence in the school generally, a facilitating influence on work processes, and/or a facilitating influence on implementation efforts. • Interviewees made general statements of how the construct manifested positively without concrete examples; and/or • There is a mixed effect of different aspects of the construct with an overall positive effect; and/or • Sufficient information exists to indirectly infer a generally positive influence.
+ 2	• The construct is a positive influence in the school generally, a facilitating influence on work processes, and/or a facilitating influence on implementation efforts. • Two or more interviewees described explicit examples of how aspects of the construct manifested positively.
Missing	• Interviewees were not asked about, or did not address, the construct in sufficient detail to discern a pattern.

^a^Adapted from Damschroder and Lowery 2013

We then employed a cross-case comparison of constructs for each school to identify constructs that most strongly influenced implementation in either a negative or positive direction as previously described [12]. We compared and contrasted CFIR construct ratings between low and high implementation schools to identify relationships between constructs and the TRUST implementation effectiveness, using data from the intermediate school to provide supporting information. We characterized each construct as not distinguishing, weakly distinguishing, or strongly distinguishing between low versus high implementation schools. We then used the detail from summary memo excerpts to assess how each construct manifested in low and high implementation schools. These findings informed our assessment of which constructs are relevant in the implementation effectiveness of participatory interventions. All included excerpts employ gender neutral language (i.e., they, their) for both TRUST core team members and school leadership to provide an additional measure of anonymity.

## Results

Of the 30 constructs we used to compare TRUST implementation barriers and facilitators, ten constructs distinguished between high and low implementation schools in TRUST, with five strongly distinguishing (see Table 4 for CFIR construct ratings by school to guide interpretation throughout the results). Below we discuss the ten distinguishing and three non-distinguishing constructs that school administrators, core researchers, and PAR researchers portrayed as influential in the implementation of this participatory trial. These constructs fell into three overarching themes – leadership engagement, alignment between the intervention and institutional priorities and systems, and tensions between adaptability and complexity within participatory interventions – and span five CFIR domains.

**Table 4:** CFIR construct ratings by school

Implementation level	Low		Intermediate	High		Distinguishing classification
School ID	1	2	3	4	5	
I. Intervention characteristics						
**Adaptability**	+ 1	+ 1	+ 1	+ 1	+ 1	
**Complexity**^a^	Missing	Missing	Missing	**−2**	**−2**	**I**
General Intervention Logistic Complexity	0	**−2**	−1	**+ 2**	0 (mixed)	
**Design Quality & Packaging**	Missing	Missing	Missing	−1	0 (mixed)	**I**
II. Outer setting						
**Participant Needs & Resources**	**−2**	−1	+ 1	+ 1	+ 1	**
**Cosmopolitanism**	Missing	0 (mixed)	+ 1	+ 1	**+ 2**	**
**External Policy & Incentives**	+ 1	Missing	0 (mixed)	0 (mixed)	+ 1	
III. Inner setting						
**Social Structural Characteristics**	**−2**	**−2**	+ 1	0 (mixed)	+ 1	*
**Networks & Communications**						
Quality of Formal Communications	0 (mixed)	−1	0 (mixed)	0 (mixed)	0 (mixed)	
Social Capital	**+ 2**	−1	+ 1	+ 1	+ 1	
**Culture**	−1	−1	+ 1	+ 1	+ 1	*
**Implementation Climate**						
Tension for Change	**−2**	−1	0 (mixed)	**+ 2**	+ 1	**
Compatibility	+ 1	+ 1	**+ 2**	**+ 2**	**+ 2**	*
Relative Priority	Missing	−1	−1	Missing	−1	
Organizational Incentives & Rewards	Missing	Missing	Missing	Missing	+ 1	**I**
**Readiness for Implementation**						
Leadership Engagement	−1	−1	+ 1	**+ 2**	+ 1	**
Available Resources	+ 1	−1	0 (mixed)	0 (mixed)	−1	
Access to Information & Knowledge	Missing	−1	+ 1	+ 1	0 (mixed)	*
IV. Characteristics of individuals						
**Knowledge & Beliefs About the Intervention**	Missing	−1	+ 1	**+ 2**	0 (mixed)	
**Agency**	−1	− 1	Missing	0 (mixed)	**−2**	
**Individual Stage of Change**	Missing	0 (mixed)	Missing	**+ 2**	**+ 2**	*
**Individual Identification with the Organization**	Missing	Missing	Missing	Missing	+ 1	**I**
V. Process						
**Planning**	0 (mixed)	−1	+ 1	−1	+ 1	
**Engaging**						
Opinion Leaders	−1	Missing	Missing	Missing	Missing	**I**
Formally Appointed Internal Implementation Leaders	**+ 2**	−1	+ 1	+ 1	+ 1	
Champions	0 (mixed)	−1	0 (mixed)	0 (mixed)	0 (mixed)	
External Change Agents	+ 1	**−2**	0 (mixed)	+ 1	+ 1	
**Executing**	0 (mixed)	**−2**	0 (mixed)	0 (mixed)	0 (mixed)	
**Reflecting & Evaluating**	Missing	**−2**	+ 1	**+ 2**	+ 1	**

Key: Rating + or - indicates the valence of the construct on implementation, and 1 or 2 indicates the magnitude of the construct’s influence on implementation

^**^ Strongly distinguishing construct

^*^ Weakly distinguishing construct

^**I**^ = Insufficient data to assess

^a^Construct is reverse rated (i.e., a positive rating denotes less complexity and vice versa)

### Outer setting

#### Participant needs and resources

This construct strongly distinguished between low and high implementation schools. PAR researchers and TRUST team members voiced examples of times when high implementation school leaders did not appear attuned to, or seemed dismissive of, student needs and preferences. Yet most reports from high implementation schools praised administrator efforts to engage student and parent perspectives in implementation, as exemplified by one leadership team’s partnership with YPAR researchers to reform in-school suspension practices. In contrast, PAR researchers and TRUST team members at the low implementation schools consistently described perceptions that students did not feel heard by adults in their schools:

‘I think that the kids [at School #2] felt that students were disconnected and that they don't necessarily understand why the adults react the way they do to some of the issues and concerns. And so, that feels really disconnecting, whether that's bullying – like, why don't you notice or why don't you do something about it? ...Why does my voice not matter?’ (TRUST YPAR Facilitator 1)

This excerpt exemplifies how differences in perceived school administrator level of awareness of student needs and preferences ultimately influenced the quality of their working relationships with PAR researchers.

#### Cosmopolitanism

Cosmopolitanism was a strongly distinguishing construct. Relationships between school leadership teams at the intermediate and high implementation schools helped to bolster implementation through positive social pressure and the sharing of resources and experiences that enhanced leadership engagement; conversely, administrators at the low implementation school did not realize their goals of building strong connections with other participating schools. One high implementation school principal reported how relationships with a previous TRUST participating school, along with their own previous experiences partnering with another university-led intervention, helped to strengthen their implementation planning. This same high implementation school also boosted another TRUST school’s interest in implementation by sharing planning materials and inviting staff to an “Intentional Social Interaction” [28] that was developed as a forum for addressing PPAR action recommendations.

### Inner setting

#### Social structural characteristics

This construct weakly distinguished between high and low implementation schools, primarily by influencing leadership engagement. Within low implementation schools, social structural characteristics such as student demographics and administrative turnover negatively influenced TRUST implementation. One low implementation school leadership team resisted implementing YPAR recommendations that did not align with the school’s demographic culturally specific mission. The other school underwent a high degree of administrative turnover resulting in fluctuating institutional goals and a hierarchical decision-making approach that made it difficult for PAR researchers and collaborators in the school to take initiative in implementation activities. Within high implementation schools, student demographics exhibited a generally positive influence on leadership responses to youth and parent recommendations. One high implementation school’s mixed score, however, reflects tensions in the effect of administrative turnover on implementation between the arrival of an enthusiastic assistant principal who closely partnered with TRUST on YPAR and this administrator’s unfamiliarity with TRUST’s other project components in their school.

#### Culture

Culture was a weakly distinguishing construct. School leaders and TRUST team members described a culture that valued student voice and exhibited openness to student leadership at one high implementation school and parent and community engagement at the other. These leaders’ orientation to student and parent voices, respectively, enabled them to build positive working relationships with PAR researchers and strengthened their commitment to implementing these TRUST components. Aspects of school culture in both low implementation schools, in contrast, contributed to implementation challenges regarding action recommendations. For example, a mismatch between YPAR recommendations and a culturally specific mission at one low implementation school contributed to a perception that this school’s administrators were dismissive of student voice (see overlap with participant needs and resources).

#### Tension for change

This construct strongly distinguished between high and low implementation schools. School administrators at the low implementation schools generally exhibited resistance to issues raised by action researchers, as one principal exemplified by dismissing student researcher concerns as irrelevant:

‘We had a hard time getting [the youth] connected with [School #2 principal]. But then, [they were] like, “Oh, but things are so much better here this year, so we don't need exactly the same things, but yes, we're really interested in seeing them.” Like, you got a middle school, you're always going to need stuff on bullying.’ (TRUST YPAR Facilitator 1)

Leadership at the high implementation schools exhibited an overall openness to change in their responses to either youth or parent researcher recommendations. One high implementation school principal displayed an openness to act on PPAR findings and recommendations but conveyed dismissiveness regarding YPAR concerns, while administrators at the other school readily partnered with YPAR researchers to establish a planning committee to implement student-led changes to in-school suspension practices.

#### Compatibility

Compatibility was a weakly distinguishing construct. Where it existed, compatibility between school priorities and PAR recommendations largely facilitated implementation through access to resources and by boosting leadership engagement. For example, one high implementation school’s prioritization of parent engagement compelled the principal’s leadership in “Intentional Social Interactions” while youth recommendations to reform in-school suspension aligned with the other high implementation school’s interest in this area. Conversely, low implementation school leaders funneled youth and parent recommendations toward existing school initiatives rather than allowing youth and parent-driven calls for change to inform a tailored or innovative response:

‘At [School #2] it sounded like, well, these are the things that we're going to do...I mean, on the one hand, it's good to align the recommendations with the things, but how do you also let what you're doing be informed by what the youth have said, right? And so, I mean it felt very much like business as usual.’ [Project TRUST YPAR Facilitator 1]

This excerpt illustrates the key role of school leadership in imagining how to align recommendations from youth to school initiatives and therefore strong project implementation.

#### Leadership engagement

However, the construct of leadership engagement strongly distinguished between high and low implementation schools. Leadership at both high implementation schools and the intermediate school maintained a high level of involvement with one or more TRUST components, which they demonstrated by prioritizing time for implementation team meetings and displaying a high level of accountability in the planning and implementation phases. At one high implementation school, for instance, the assistant principal actively supported YPAR recommendation implementation through regular meetings and advocacy:

‘I met with the students every other week…And they were really good about saying, “How’s this coming along? How do you see this working? Why isn’t this done yet?”...We were very upfront and honest about why things aren’t moving along faster.’ (School #4 Assistant Principal)

Lower levels of involvement and accountability among leadership distinguished the two low implementation schools. At one school, TRUST team members identified early on that the primary point of contact for the parent researchers, who joined mid-project, had poor buy-in to the project’s goals and a negative attitude that limited their engagement. Though the principal at the other school initially exhibited a high level of engagement, they lacked follow-through on TRUST commitments as the year progressed. When asked about this change, the principal responded that school improvements no longer warranted responding to YPAR recommendations, highlighting the overlap between leadership engagement and tension for change.

#### Access to information and knowledge

The ease of school members’ access to information and knowledge about TRUST weakly distinguished between high and low implementation schools. The TRUST team had more consistent opportunities to disseminate project details among staff at both high implementation schools. One high implementation school invited TRUST to conduct a PD session while simultaneously sharing PAR research findings and recommendations. The mixed score for the other high implementation school reflects low attendance at the staff meeting to share information about TRUST counterbalanced by the influential teachers who attended:

‘Only two teachers came...But they were two teachers who have weight in the schools, one the student council rep and the other one also seemed to be in a position of how to get students more voice. They gave ideas for how kids can be more involved in the schools in interventions and evaluation.’ (TRUST YPAR Facilitator 2)

### Characteristics of individuals

#### Individual stage of change

This construct weakly distinguished implementation among the three schools with enough data. Leaders within the two high implementation schools demonstrated a consistent readiness for change, positive engagement with recommendations and involvement in implementation activities. In contrast, the disposition of one low implementation school principal changed over the implementation year from enthusiastic to questioning the relevance of TRUST for their school.

### Process

#### Reflecting and evaluating

This was a strongly distinguishing construct. One low implementation school provided limited opportunities for reflection and evaluation, whereas both high implementation schools demonstrated the value of making time for these activities to iteratively inform the next stages of the work, particularly for the PAR recommendations. For example, one high implementation school’s reflections on implementing PAR recommendations highlighted challenges with engaging parent researchers:

‘[School #5 parent researcher was] really involved with planning the IZI, but feels out of the loop with what comes next…[They weren’t] able to attend the debriefing meeting due to [their] schedule. [but they] sent me extensive notes about what we should have done differently and next steps, and I shared these during the meeting on [their] behalf. When [the parent researcher] saw this reflected in the notes, I think [they] felt better...But then, the issue still remains around what is the role of the parent researcher in making those action steps happen. What should [they] be doing? Who should [they] be connecting with?’ (TRUST PPAR Facilitator 1)

### Non-distinguishing constructs

Several constructs, though non-distinguishing, nonetheless played an influential role in this participatory intervention. Adaptability manifested as a weakly positive influence in all schools. While the inherent adaptability of TRUST’s approach in which PAR researchers developed unique recommendations for each school appealed to many school leaders, this adaptability also caused confusion among administrators about the relationships between TRUST’s components, how they related to the intervention’s overall goals, and expectations for moving implementation forward. One principal explicitly describes this confusion:

‘What I need to know is what’s expected. Just number one, two, and three. Component one, component two, component three, so we know exactly what we’re talking about. Because...I understand the focus of the grant, but what does that mean?’ (School #5 Principal)

This principal’s comment highlights the relationship between complexity and design quality and packaging. Although we lacked data from the low implementation schools to assess these latter two constructs, our experiences implementing TRUST highlight their relevance for participatory intervention implementation. For example, leaders at the high implementation schools described complexity as stemming from the lack of previously delineated implementation plans and expectations of school leadership involvement at the project’s onset (design quality and packaging), and they expressed frustration with the frequent intervention iterations and the resulting confusion about the relationship between TRUST’s multiple components — all challenges related to the participatory approach that likely hindered TRUST’s implementation.

Schools also appeared to struggle with agency, a construct adapted to capture the broader contextual influences on an individual’s self-efficacy (Table 2). One high implementation school principal attributed their struggle to engage parents of color (a PAR recommendation) to structural challenges, including a lack of a parent engagement coordinator and parent-teacher organization meeting times that often precluded many parents of color from attending.

Finally, while none of the four engaging sub-constructs distinguished between high and low implementation schools, these roles underscored how TRUST team member roles spanned categories and blurred the boundaries between internal and external school affiliations. For example, one TRUST team member, who also worked within the school district, filled roles that we classified at various points in the analysis as opinion leader, formally appointed implementation leader, and champion.

## Discussion

This study represents one of the first applications of the CFIR within a participatory trial, Project TRUST, and thus provided a unique opportunity to examine how the CFIR functions within a participatory implementation context. Here we highlight ten constructs that distinguished between high and low implementation schools and three additional constructs that played influential, though non-distinguishing, roles in this multi-component participatory trial. Relevant CFIR constructs fell into three broad themes that spanned the five CFIR domains.

First, administrators’ level of leadership engagement emerged as a key distinguishing construct and an overarching theme of important influences on TRUST’s implementation. Administrators in schools with higher implementation generally demonstrated higher levels of commitment, involvement, and accountability with implementation activities. Leadership engagement is important in the implementation of any intervention [12, 17], but especially within participatory interventions, which demand high levels of active collaboration relative to conventional interventions where leaders may play a more distant role [34]. As our findings indicate, collaboration within a participatory intervention demands consistent and strong communication and negotiation between leadership and other stakeholders [6, 7] and involvement of leaders in the day-to-day implementation processes [21].

Several constructs enhanced leadership engagement as a means of promoting implementation. For example, leaders who exhibited an awareness of the needs and preferences of their student bodies (participant needs and resources) and who described their school culture as more consistently welcoming to student and parent voices formed stronger partnerships with PAR researchers and maintained a higher level of commitment to implementing the resulting action recommendations. Though previous research has indicated that a welcoming organizational culture is not essential to the implementation of conventional interventions [35], other participatory interventions have highlighted the importance of cultural openness to effective implementation [7, 36]. Furthermore, while tension for change is a frequently cited influential implementation construct more broadly [12, 37], we and others have observed that leadership openness to identifying stakeholder-raised issues as problematic and their desire to partner with these stakeholders to develop solutions directly influences their level of engagement and is therefore especially important within participatory interventions [3, 7]. Participatory interventions also demand high levels of innovation and flexibility from institutional leaders [8] that can stretch a leader’s perceived capacity, potentially lowering their engagement. Finally, we found that higher levels of support from administrative peers at other institutions (cosmopolitanism), a previously highlighted influential construct for implementation within school settings [17], enhanced leadership engagement by exerting social pressure and sharing resources and experiences that informed new collaborations and implementation modalities such as the Intentional Social Interactions. Conversely, administrative turnover emerged as a potential negative influence on TRUST’s implementation, particularly for a school with low scores across many constructs, when it reduced leadership familiarity with project components, altered institutional goals, or resulted in greater hierarchy in decision-making – all outcomes that are likely to translate to participatory implementation challenges.

The degree of alignment between TRUST and existing school programs and systems was a second cross-cutting theme. Schools with weaker alignment between their student body demographics (social structural characteristics) and their cultural mission experienced more challenges in recognizing and responding to PAR recommendations. Additionally, TRUST implementation was higher when existing school workflows, systems, and leadership beliefs closely aligned with the proposed action recommendations (compatibility) as previous participatory interventions implemented in school settings have observed [3]. Consideration of alignment with school systems is particularly important for participatory interventions, in which incorporating stakeholder involvement throughout the research process may preclude pre-defining intervention components and necessary resources at the beginning of a collaboration [21]. Early institutional-intervention alignment can enhance leadership buy-in at a time when discrete interventions components have yet to be established [36]. However, while aligning intervention components with school systems can facilitate implementation, our experiences with TRUST suggest that it may do so at the expense of authentic leadership engagement with stakeholder-voiced issues and proposed solutions within participatory collaborations.

Finally, we observed an oppositional relationship between two non-distinguishing constructs, adaptability and complexity**,** which provides insights into how these constructs may function in participatory interventions more broadly. While adaptability is often cited as an implementation strength of the participatory approach because it provides for enhanced individualization to meet the unique needs of different contexts [3, 7, 19], the iterative nature of TRUST’s PAR components appeared to contribute to school leaders’ confusion and influenced perceptions of TRUST as a complex intervention, as evidenced by several administrators’ frustration with the lack of pre-defined implementation plans at the project’s onset. Our findings underscore the importance of preempting stakeholder frustration with the iterative process of participatory trials at the beginning of the collaboration by articulating, as Hawe et al. describe, that potential implementation avenues (or the “steps in the change process”) are the standard aspects of the intervention rather than specific components or programs [38]. Leadership engagement across implementation stages may ameliorate confusion stemming from perceived complexity of participatory intervention implementation [36]. Further examination of how these constructs should be adapted for participatory intervention is warranted.

The CFIR initially conceptualized interventions as unidirectional transfers of discrete components according to an implementation plan from an external developer to a recipient organization [11]. In this paradigm, implementation team roles tended to be distinct with clearly defined “outsiders” and “insiders” to the implementation sites. Our analysis highlighted two challenges in adapting this paradigm to a participatory intervention. First, TRUST implementation team members generally spanned roles within the engaging construct. The involvement of student and parent stakeholders further blurred the lines between implementation roles. Furthermore, participatory interventions are co-developed and co-implemented by stakeholders with varying levels of connection to an institution as previously described [5]. The dynamic and collaborative nature of participatory intervention implementation thus challenges the binaries of those who are internal or external to an organization (and the related distinctions in the CFIR’s inner and outer setting domains, which are often overlapping in participatory research). Rather, participatory interventions’ involvement of stakeholders from across different spheres of influences can enhance buy-in among a diverse group of individuals in ways that strengthen the implementation process and intervention sustainability [7].

The second challenge in adapting the CFIR to a participatory implementation process relates to the framework’s focus on individual behavior changes. Although considering how an individual’s changing beliefs toward the intervention and their degree of identification with the organization is important, assessments of participatory intervention implementation must also include an evaluation of how dynamic interactions between stakeholders at multiple levels of the implementation shape their relationships and, in turn, the implementation process itself [38]. For example, we observed a longitudinal decrease in one principal’s engagement with TRUST at one low implementation school (as described in the leadership engagement construct) that stemmed directly from a deteriorating quality of communication and misaligned expectations within the partnership. While this idea is implicit within several constructs (e.g., reflecting and evaluating, which strongly distinguished between high and low implementation in our analysis), assessments of participatory intervention implementations would benefit from a more explicit examination of how group interactions longitudinally influence the implementation process. In our analysis, we also adapted the construct self-efficacy to agency to highlight the shift from an individual-focused construct to one that is shaped by contextual factors such as relationships and system-level factors inherent to participatory interventions. Similar adaptations of other individual-focused constructs will increase the fit of the CFIR within a participatory context.

### Limitations

This study has several limitations to consider. First, our qualitative data emphasize the perspectives of TRUST implementation team members and school administrators from participating schools. While we included a wide range of perspectives including academic and community researchers, school district collaborators and school administrators, and PAR researchers, our data from PAR researchers and administrators was more limited. Our analysis may therefore not have captured the full range of these stakeholders’ experiences. Second, while we examined constructs that spanned the CFIR domains, we lacked adequate data to assess all of the constructs that we initially deemed relevant for TRUST implementation. Third, we encountered several methodologic challenges in adapting the analytical approach to a longitudinal implementation assessment of a multi-component intervention. Longitudinal data sources enabled us to see changes in how constructs operated within a school over time, which made applying one rating to the construct challenging. We elected to average construct ratings when we noted significant changes over time; however, this approach may have neutralized the more extreme effects of some constructs and influenced our final interpretation of their respective influences on implementation. Additionally, we aimed to produce a global assessment of each construct across TRUST’s three components. This approach may have oversimplified the influence of specific constructs on one or more intervention components, particularly in cases where the construct had a negative influence on one component and a positive influence on another. Finally, our data represent the experiences of implementing one participatory research intervention in five schools in one urban school district and may not be widely generalizable.

## Conclusions

Our findings support the CFIR as a useful framework in assessing the implementation of participatory research trials, an understudied area within implementation science. Constructs from across the five domains of the CFIR aided in our assessment of participatory intervention implementation effectiveness, but particularly those related to leadership engagement and alignment between the intervention and institutional goals, priorities, demographics, and existing systems. This analysis also highlighted a tension between the benefits of a participatory intervention’s adaptability and its perceptions of complexity that should be considered when designing implementation approaches for these types of interventions. Our findings further suggest potential adaptations of the CFIR to a participatory research context that might strengthen its utility as a tool to systematically assess the implementation of participatory research trials.

## Supplementary Information

**Additional file 1.** Project TRUST team member regular semi-structured interview guiding questions.

**Additional file 2.** Project TRUST school administrator interview question guide.

## Data Availability

Deidentified qualitative datasets analyzed during the current study are available from the corresponding author on reasonable request.

## Abbreviations

CFIR: Consolidated Framework for Implementation Research
YPAR: Youth participatory action research
PPAR: Parent participatory action research
PAR: Participatory action research
PD: Professional development
TRUST: Project TRUST (Training for Resiliency in Urban Students and Teachers)
BIPOC: Black, Indigenous, People of Color

## References

[1] Izumi BT, Peden AM, Hallman JA, Barberis D, Stott B, Nimz S, Ries WR, Capello A (2013). A community-based participatory research approach to developing the harvest for healthy kids curriculum. Prog Community Heal Partnerships Res Educ Action.

[2] Lindquist-Grantz R, Abraczinskas M (2020). Using youth participatory action research as a health intervention in community settings. Health Promot Pract.

[3] Okamoto SK, Helm S, Chin SK, Hata J, Hata E, Okamura KH (2020). The implementation of a culturally grounded, school-based, drug prevention curriculum in rural Hawai‘i. J Community Psychol.

[4] Schelvis RMC, Wiezer NM, Blatter BM, Van Genabeek JAGM, Oude Hengel KM, Bohlmeijer ET (2016). Evaluating the implementation process of a participatory organizational level occupational health intervention in schools. BMC Public Health.

[5] Breimaier HE, Heckemann B, Halfens RJG, Lohrmann C (2015). The consolidated framework for implementation research (CFIR): a useful theoretical framework for guiding and evaluating a guideline implementation process in a hospital-based nursing practice. BMC Nurs.

[6] Morgan D, Kosteniuk J, O’Connell ME, Kirk A, Stewart NJ, Seitz D (2019). Barriers and facilitators to development and implementation of a rural primary health care intervention for dementia: a process evaluation. BMC Health Serv Res.

[7] Warren CE, Ndwiga C, Sripad P, Medich M, Njeru A, Maranga A, Odhiambo G, Abuya T (2017). Sowing the seeds of transformative practice to actualize women’s rights to respectful maternity care: reflections from Kenya using the consolidated framework for implementation research. BMC Womens Health.

[8] Allen ML, Garcia-Huidobro D, Bastian T, Hurtado GA, Linares R, Svetaz MV (2017). Reconciling research and community priorities in participatory trials: application to padres Informados/Jovenes Preparados. Fam Pract.

[9] Nilsen P (2015). Making sense of implementation theories, models and frameworks. Implement Sci.

[10] CFIR Research Team-Center for Clinical Management Research. Consolidated Framework for Implementation Research [Internet]. 2020. [cited 2020 Apr 8]. Available from: https://cfirguide.org/.

[11] Damschroder LJ, Aron DC, Keith RE, Kirsh SR, Alexander JA, Lowery JC (2009). Fostering implementation of health services research findings into practice: a consolidated framework for advancing implementation science. Implement Sci.

[12] Damschroder LJ, Lowery JC (2013). Evaluation of a large-scale weight management program using the consolidated framework for implementation research (CFIR). Implement Sci.

[13] Keith RE, Crosson JC, O ‘Malley AS, Cromp D, Taylor EF (2017). Using the Consolidated Framework for Implementation Research (CFIR) to produce actionable findings: a rapid-cycle evaluation approach to improving implementation. Implement Sci.

[14] Ware P, Ross HJ, Cafazzo JA, Laporte A, Gordon K, Seto E (2018). Evaluating the implementation of a mobile phone-based telemonitoring program: longitudinal study guided by the consolidated framework for implementation research. JMIR mHealth uHealth.

[15] Northridge ME, Kavathe R, Zanowiak J, Wyatt L, Singh H, Islam N (2017). Implementation and dissemination of the Sikh American families Oral health promotion program. Transl Behav Med.

[16] Hudson KG, Lawton R, Hugh-Jones S (2020). Factors affecting the implementation of a whole school mindfulness program: a qualitative study using the consolidated framework for implementation research. BMC Health Serv Res.

[17] Leung E, Wanner KJ, Senter L, Brown A, Middleton D (2020). What will it take? Using an implementation research framework to identify facilitators and barriers in implementing a school-based referral system for sexual health services. Health Serv Res.

[18] Fernandez ME, Walker TJ, Weiner BJ, Calo WA, Liang S, Risendal B, Friedman DB, Tu SP, Williams RS, Jacobs S, Herrmann AK, Kegler MC (2018). Developing measures to assess constructs from the inner setting domain of the consolidated framework for implementation research. Implement Sci.

[19] Ramanadhan S, Davis MM, Armstrong R, Baquero B, Ko LK, Leng JC, Salloum RG, Vaughn NA, Brownson RC (2018). Participatory implementation science to increase the impact of evidence-based cancer prevention and control. Cancer Causes Control.

[20] Hawe P, Shiell A, Riley T (2004). Complex interventions: how “out of control” can a randomised controlled trial be?. Br Med J.

[21] Dias S, Gama A, Simões D, Mendão L (2018). Implementation process and impacts of a participatory HIV research project with key populations. Biomed Res Int.

[22] Bond L, Butler H, Thomas L, Carlin J, Glover S, Bowes G (2007). Social and school connectedness in early secondary school as predictors of late teenage substance use, mental health, and academic outcomes. J Adolesc Heal.

[23] Weatherson KA, O’Neill M, Lau EY, Qian W, Leatherdale ST, Faulkner GEJ (2018). The protective effects of school connectedness on substance use and physical activity. J Adolesc Health.

[24] Klem AM, Connell JP (2004). Relationships matter: linking teacher support to student engagement and achievement. J Sch Health.

[25] Bottiani JH, Bradshaw CP, Mendelson T (2014). Promoting an equitable and supportive school climate in high schools: the role of school organizational health and staff burnout. J Sch Psychol.

[26] Voight A, Hanson T, O’Malley M, Adekanye L. The racial school climate gap: within-school disparities in students’ experiences of safety, support, and connectedness. Am J Community Psychol. 2015;56(3-4):252–67. 10.1007/s10464-015-9751-x. 10.1007/s10464-015-9751-x 26377419

[27] Peguero AA, Bondy JM (2011). Immigration and students’ relationship with teachers. Educ Urban Soc.

[28] Marnita’s Table [Internet]. 2020. [cited 2020 Apr 8]. Available from: https://projects.marnitastable.org/.

[29] O’Brien BC, Harris IB, Beckman TJ, Reed DA, Cook DA (2014). Standards for reporting qualitative research: a synthesis of recommendations.

[30] Hsieh H-F, Shannon SE (2005). Three approaches to qualitative content analysis. Qual Health Res.

[31] Ahearn L (2001). Language and agency. Annu Rev Anthropol.

[32] Dedoose (2018). Web application for managing, analyzing, and presenting qualitative and mixed method research data.

[33] Hill CE, Thompson BJ, Hess SA, Knox S, Williams EN, Ladany N (2005). Consensual qualitative research: an update. J Couns Psychol.

[34] Varsi C, Ekstedt M, Gammon D, Ruland CM (2015). Using the consolidated framework for implementation research to identify barriers and facilitators for the implementation of an internet-based patient-provider communication service in five settings: a qualitative study. J Med Internet Res.

[35] Cannon JS, Gilbert M, Ebener P, Malone PS, Reardon CM, Acosta J, Chinman M (2019). Influence of an implementation support intervention on barriers and facilitators to delivery of a substance use prevention program. Prev Sci.

[36] Fish J (2016). Co-producing knowledge about lesbian and bisexual women with breast cancer: messages for nursing professionals from a knowledge exchange project. J Res Nurs.

[37] King DK, Shoup JA, Raebel MA, Anderson CB, Wagner NM, Ritzwoller DP, Bender BG (2020). Planning for implementation success using RE-AIM and CFIR frameworks: a qualitative study. Front Public Heal.

[38] Hawe P, Bond L, Butler H, Ottoson JM, Hawe P (2009). Knowledge theories can inform evaluation practice: What can a complexity lens add?. Knowledge utilization, diffusion, implementation, transfer, and translation: Implications for evaluation New Directions for Evaluation.

