# Supplementary material for: Evaluation of a school-based participatory intervention to improve school environments using the Consolidated Framework for Implementation Research
Source: BMC Public Health. 2021 Sep 3;21:1615. doi: 10.1186/s12889-021-11644-5 (PMC8414723; doi:10.1186/s12889-021-11644-5)
Supplement: Supplementary file 1 — Additional file 1. Project TRUST team member regular semi-structured interview guiding questions. [file 12889_2021_11644_MOESM1_ESM.docx]

**Supplementary File 1**

**Project TRUST team member regular semi-structured interview guiding questions**

1. Describe recent project implementation activities that you have been involved with since we last spoke.
2. What is going well with the project implementation?
   1. Teacher Professional Development
   2. Parent Participatory Action Research (PPAR)
   3. Youth Participatory Action Research (YPAR)
3. What challenges have you encountered with project implementation?
   1. Teacher Professional Development
   2. PPAR
   3. YPAR
4. What has surprised you about your work implementing TRUST in the past few weeks?
5. Please share any overall reflections on your experiences carrying out the implementation tasks.
6. Please share any overall reflections on the TRUST partnership as it influences implementation.
