# Supplementary material for: Evaluation of a school-based participatory intervention to improve school environments using the Consolidated Framework for Implementation Research
Source: BMC Public Health. 2021 Sep 3;21:1615. doi: 10.1186/s12889-021-11644-5 (PMC8414723; doi:10.1186/s12889-021-11644-5)
Supplement: Supplementary file 2 — Additional file 2. Project TRUST school administrator interview question guide. [file 12889_2021_11644_MOESM2_ESM.docx]

**Supplementary File 2**

**Project TRUST school administrator interview question guide**

1. Can you tell us how you initially became involved with TRUST? Probes: How did you first hear about TRUST? What convinced you to become involved? What convinced you that TRUST might work (evidence of intervention)? How did it align with other district priorities?
2. We know that you have many initiatives in your school. What were your highest priorities over the last 2 years? Which of these were focused on climate or connectedness? How did TRUST fit within these priorities, particularly for climate and connectedness, both connecting to students and parents?
3. How do you perceive student-school connectedness in your school? Can you give us some examples? (Clarification: we are thinking about which groups have an easier time being connected and for which is it harder and why). How has your school’s involvement in TRUST activities helped shape youth and parent school connectedness? Can you give us some examples?
4. What are two or three of the most important core values at your school? How did those values generally help or hinder the implementation of TRUST? (have a few examples if they are struggling)

**Now let’s talk about your experience with TRUST in your school**

1. What was the easiest aspect of TRUST to implement? What was the most difficult or complicated aspect? (Probe for answers related to Youth Participatory Action Research, Parent Participatory Action Research, and Teacher Professional Development). What were some of the barriers to using the Teacher Professional Development specifically?

Clarification: when we’re talking about implementation, we’re talking about recruiting parents and youth, helping them do their research in school, using the PD (year 1) and making use of the parent and youth recommendations (year 2).

1. Who do you consider your internal TRUST team and what are their roles in the school? How would you describe the working relationships between these people? How well did these people communicate? What was most challenging? What worked well?
2. How did TRUST fit within the work and systems currently in place in your school to address connectedness? (might have been addressed in Q.3)
3. In what ways were TRUST activities and staff adaptable to your school’s needs? Were there downsides to adaptability/flexibility? (Give more context as needed). Were there aspects of TRUST were more structure would have been helpful?

**Now let’s talk about everybody who have been involved with TRUST (external teams).**

1. In what ways did the TRUST team provide your desired level of support for TRUST implementation? How could the TRUST team have been more supportive in each of the three components (Youth Participatory Action Research, Parent Participatory Action Research, Teacher Professional Development)?
2. In what ways did TRUST provide sufficient resources for program activities? In what areas were more resources needed?
3. In what ways did you have the resources and support to sustain Project TRUST activities?

**Review of the Youth and Parent Participatory Action Research recommendations**

1. Review of the grid of youth and parent researcher action recommendations (one-by-one) with a guided reflection on the school’s implementation approach and their self-assessments related to this.
   1. What has happened based on each recommendation?
   2. What else did we miss in this grid?
   3. What else has happened because of TRUST?
